# Bacteriological Profile and Antibiogram Pattern of Wound Infection in a Tertiary Care Hospital

**DOI:** 10.7759/cureus.72185

**Published:** 2024-10-23

**Authors:** Sejal R Bhujugade, Geeta Karande, Satish Patil

**Affiliations:** 1 Department of Microbiology, Krishna Institute of Medical Sciences, Krishna Vishwa Vidyapeeth (Deemed to be University), Karad, IND

**Keywords:** antibiotic resistance, antibiotic susceptibility, gram-negative bacteria, gram-positive bacteria, klebsiella spp, staphylococcus aureus, wound infection

## Abstract

Background and aim

Wound infection constitutes a major barrier to healing and can have a harmful effect on the patient’s quality of life and also on the healing rate of the wound. The widespread use of antibiotics, along with their extensive history of availability, has resulted in significant issues with resistant organisms contributing to morbidity and mortality. The study was conducted to determine the prevalence of bacterial species obtained from the wound sample and its antibiotic resistance pattern.

Materials and methods

The cross-sectional observational study was conducted from November 2022 to November 2023 in a tertiary care hospital in Karad. 100 clinical samples of wound swabs and pus were studied. The isolates from these clinical samples were further processed as per the standard prescribed for the identification and antimicrobial susceptibility testing.

Results

Out of 100 isolates, 75 were gram-negative and 25 gram-positive. Among the gram-negative isolates, the predominantly isolated organism was *Klebsiella* spp. 20(26.66%), and gram-positive bacteria was mainly *Staphylococcus aureus* (23, 92%). Most of the isolates showed sensitivity to vancomycin (20, 86.95%) and resistance to penicillin (18, 78.26%). *Klebsiella* spp. (20, 26.66%) was mostly sensitive to piperacillin/tazobactam (7, 50%), and resistance was seen to ampicillin (12, 85.71%).

Conclusion

This study showed that *Staphylococcus aureus* was the most common bacterial isolate causing wound infection, followed by *Klebsiella *spp. Antimicrobial susceptibility test showed that vancomycin was effective, followed by tetracycline for *Staphylococcus aureus* and piperacillin/tazobactam, and tigecycline was effective for *Klebsiella* spp.

## Introduction

A wound occurs when the skin is physically disrupted, creating a pathway for bacterial infection in the underlying tissues. This disruption is a common factor in all types of wound trauma. Trauma may be accidental or intentionally induced [1]. It provides a moist, warm, nutritive environment conducive to microbial colonization and proliferation [2]. Infection in a wound constitutes a major barrier to healing and can have an adverse effect on the patient’s quality of life and on the healing rate of the wound [1]. Many bacterial species live on human skin, in the nasopharynx, gastrointestinal tract, and other parts of the body, with little potential to cause disease due to the first line of defense within the body [2].

This study hypothesizes that the bacteriological profile of wound infections in patients at a tertiary care hospital will disclose a wide array of pathogenic microorganisms, with corresponding antibiogram patterns exhibiting significant variability in antibiotic susceptibility based on wound type and patient demographics. Both gram-positive as well as gram-negative organisms can cause wound infection; gram-positive bacteria like coagulase-positive *Staphylococcus aureus, Enterococcus fecalis, Enterococcus faecium* etc” and gram-negative organisms like *E. coli*, *Klebsiella* spp., *Pseudomonas aeruginosa*, *Proteus* *mirabilis*, *Acinetobacter* spp., *Enterobacter,* etc [3,4]. Wound healing requires a good, healthy environment for a typical physiological process that results in minimal scars and a normal healing process. The most important strategy for healing is to sterilize damaged tissues from any microbial infection [1]. Wound infection has been a longstanding issue in the medical field. The widespread use of antibiotics for inappropriate indications and prolonged duration has led to major problems of resistant organisms, contributing to increased morbidity and mortality. Understanding the factors responsible for wound infection has proven to be helpful in the selection of appropriate antimicrobial therapy and infection control measures taken in health institutions [2].

Most hospitals in developing countries have poor and highly compromised infection programs due to limited access to clean water, poor personal hygiene practices, insufficient laboratory support, and a lack of funding. Correct information about the incidences and causative agents of infection is essential [1].

## Materials and methods

Study design, period, sample size, and data collection

The study was designed as a cross-sectional observational study to find out the bacterial profile and antibiogram pattern. The study period was from November 2022 to November 2023. The research received approval from the Ethical Committee of Krishna Vishwa Vidyapeeth, Deemed to be University, Karad, with Ethical Certificate No 066/2021-2022. 100 clinical samples of wound swabs and pus were collected and processed. The clinical specimens were collected from Krishna Hospital and Medical Research Centre (KH&MRC) and analyzed at the Department of Microbiology, Krishna Institute of Medical Sciences, Karad. The primary outcome of the current study was to isolate various bacterial agents of wound infection, and the secondary outcomes of this study encompass the evaluation of the antimicrobial susceptibility patterns of the isolated bacteria and a thorough assessment of drug resistance patterns among the identified pathogens.

Inclusion criteria

Patients of both sexes and of all age groups clinically diagnosed with wound infections other than post-operative wounds from the inpatient and outpatient departments of Krishna Hospital and Medical Research Center, Karad, were included.

Exclusion criteria

Post-operative wound samples were excluded.

Sample collection

Clinical samples were received in the Department of Microbiology from Krishna Hospital and Medical Research Centre, Karad. The clinical samples, such as pus and wound swabs from both genders and all groups of patients, were included. Samples were gathered using aseptic techniques in sterile containers. Storage and transportation of the samples were maintained in cold conditions (8-10°C) until processing.

Sample processing

Microscopy

A clean, oil-free slide was taken to identify bacteria primarily based on their microscopic observation. Upon that, a drop of normal saline (0.85%) was added, and a thin uniform smear of collected wound and pus sample was prepared. Heat fixing of the smear is done. After heat fixing the slide, the smear was stained by the Gram staining technique and observed using an oil immersion objective lens (100X) of a light microscope. The smear was checked for the presence of gram-positive and gram-negative bacteria.

Identification of Isolate

The clinical samples were inoculated onto nutrient agar, MacConkey agar, blood agar, and chocolate agar. The isolates were identified by examining colony morphology on nutrient agar, MacConkey agar, blood agar, and chocolate agar after incubating at 37˚C for 24 hours. A Gram stain was performed on smears taken from the colonies. Oxidase and catalase tests were conducted to identify the colonies. Further biochemical reactions were carried out as per the prescribed standards [5].

Phenotypic test 

Antimicrobial Susceptibility Test

**Figure 1:**
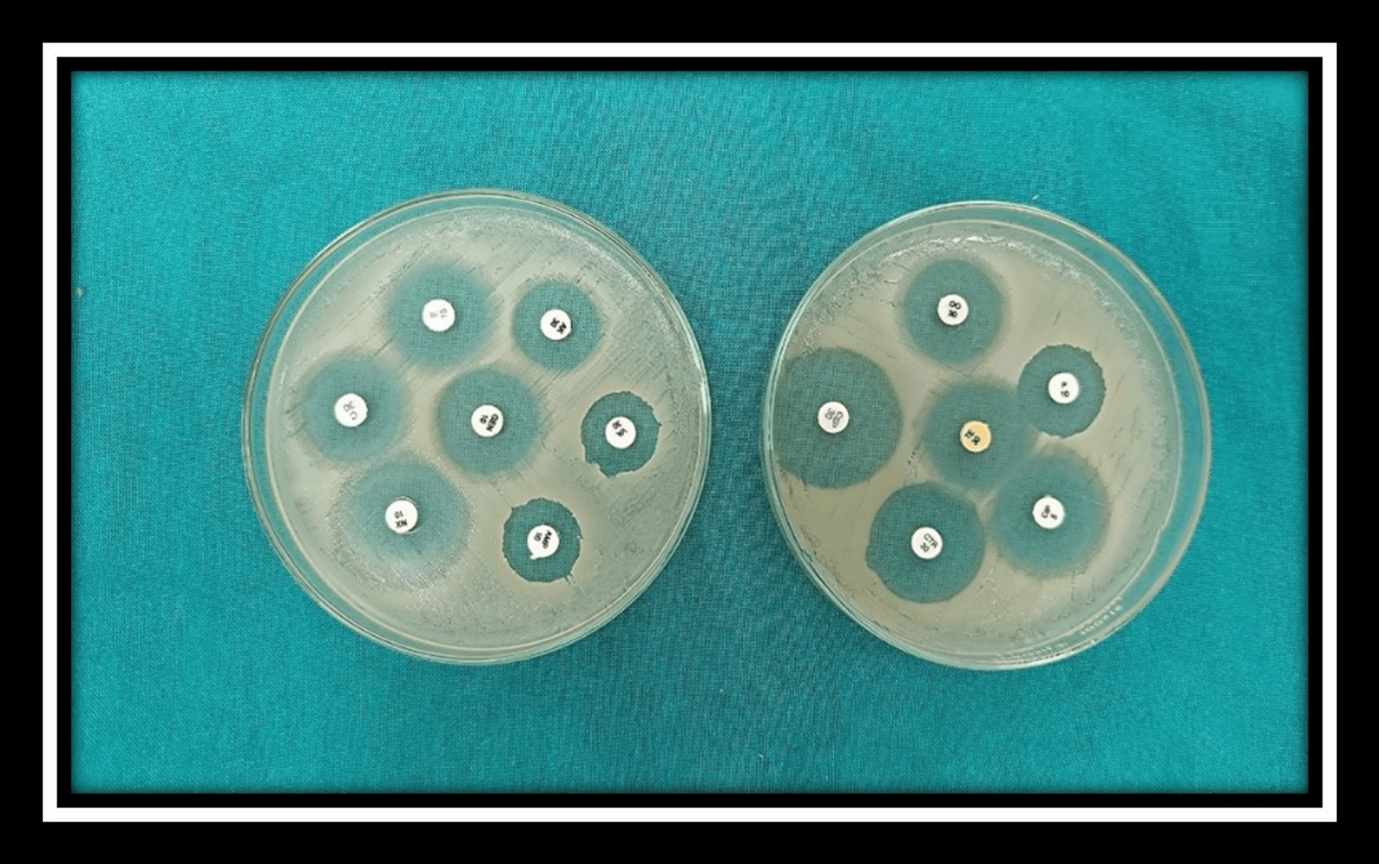
Antimicrobial susceptibility testing of gram-positive organism Gram-positive organism : *Staphylococcus aureus *on Mueller-Hinton agar Sensitive : doxycycline (DO), penicillin (P), tetracycline (TE), ciprofloxacin (CIP), gentamicin (GEN), erythromycin (E)

**Figure 2:**
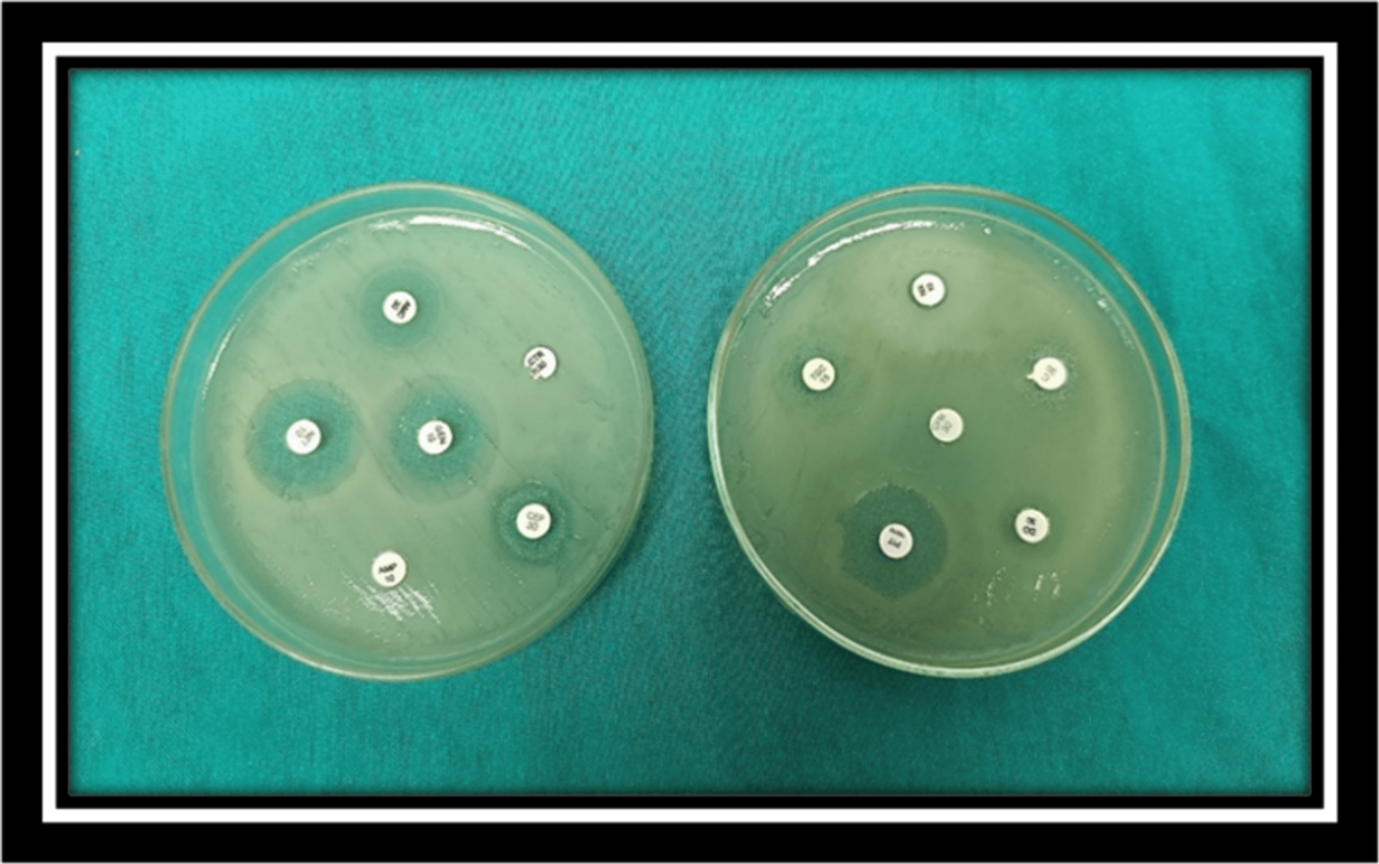
Antimicrobial susceptibility testing of gram-negative organism Gram-negative organism : *Klebsiella pneumoniae *on Mueller Hinton agar Sensitive: gentamicin (GEN), netilmicin (NET), amoxicillin/clavulanic acid (AMC)

Antimicrobial susceptibility testing of isolates was performed on Muller Hinton agar plate using the Kirby-Bauer disc diffusion method according to the clinical and laboratory standard institute (CLSI 2022). The inoculum of test and control organism suspension was prepared and matched with the optical density of 0.5% McFarland turbidity standard. Test and control organism suspension was inoculated on Muller-Hinton agar plate. After drying, the antibiotic disc was kept on lawn culture. The plate was kept for the incubation at 37°C for 16-22 hours. The inhibition zone shows that the antibiotic is sensitive to the organism. The susceptibility was tested against ampicillin (10µg), ciprofloxacin (5µg), norfloxacin (10µg), cephalothin (30µg), gentamicin (10µg), tetracycline (30µg), cotrimoxazole(25µg), chloramphenicol(30µg), doxycycline (30µg), nalidixic acid (15µg), ceftriaxone (30µg), penicillin G (10IU), erythromycin (15µg) and vancomycin (30µg) for gram-positive and gram-negative bacterial isolates (Figure 1 and Figure 2). Control strains taken are *Staphylococcus aureus* ATCC 25923, *E. coli* ATCC 25922, *Pseudomonas aeruginosa* ATCC 27853.

Statistical analysis

Data was filled in MS Excel Software. The results were expressed as percentages, and tables were generated to meet different objectives.

## Results

100 clinical samples of wounds were studied in the present study. The results of the study are given below;

Table 1 shows the age and gender-wise distribution of wound infection. The organisms isolated from the age group of 0-20 years accounted for 7%, followed by 42% in the 21-40 years age group, 28% in the 41-60 years group, and 23% in those over 60 years. In females, the highest number of isolates was observed in the 21-40 age group with 17 cases (35.55%), followed by the 41-60 age group with 12 cases (26.66%), the >60 age group with 11 cases (24.44%), and the 0-20 age group with 6 cases (13.33%). In males, the highest number of isolates was found in the 21-40 age group with 25 cases (45.45%), followed by 16 cases (30.90%) in the 41-60 age group, 12 cases (21.81%) in the >60 age group, and 1 case (1.81%) in the 0-20 age group.

**Table 1:** Age and gender wise distribution of wound infection n: Number; %: Percentage

Age group	Male, n (%)	Female, n (%)	Total, n (%)
0-20	1 (1.81)	6 (13.33)	7 (7)
21-40	25 (45.45)	17 (35.55)	42(42)
41-60	16 (30.90)	12 (26.66)	28 (28)
>60	12 (21.81)	11 (24.44)	23 (23)
Total(n)	54 (54)	46 (46)	100 (100)

Table 2 shows the location-wise distribution of wound infection. The maximum isolates were from inpatients (72%), compared to outpatients (28%).

**Table 2:** Location wise distribution of wound infection

Location	Isolation (n)	Percentage (%)
Inpatient	72	72
Outpatient	28	28
Total	100	100

Table 3 shows the gram nature of the organisms. The majority, 75%, were gram-negative, compared to gram-positive 25%.

**Table 3:** Gram nature of the organisms

Gram nature	Total isolated (n)	Percentage (%)
Gram-positive	25	25
Gram-negative	75	75
Total	100	100

Table 4 shows the distribution of wound infections from the hospital departments. The highest number of isolates came from the ICU, accounting for 20 cases (20%), followed by orthopedics with 19 cases (19%), surgery with 18 cases (18%), medicine with 16 cases (16%), gynecology and obstetrics with 13 cases (13%), neurosurgery and NICU with 5 cases each (5%), and critical care unit (CCU) and ear, nose, and throat (ENT) with 2 cases each (2%).

**Table 4:** Distribution of wound infection from the departments of hospital n: number; %: percentage; ICU: intensive care unit; Gyn & Obgy: gynecology and obstetrics; NICU: neonatal intensive care unit; CCU: critical care unit; ENT: ear, nose, and throat

Department	Samples (n)	Percentage (%)
ICU	20	20
Surgery	18	18
Neurosurgery	05	05
Gyn & Obgy	13	13
Pediatric (NICU)	05	05
Orthopedics	19	19
CCU	02	02
ENT	02	02
Medicine	16	16
Total	100	100

Table 5 shows the antibiotic susceptibility test of gram-positive organisms isolated from wound infection. *Staphylococcus aureus* showed maximum sensitivity to vancomycin (20, 86.95%) and maximum resistance to penicillin (18, 78.26%). *Enterococcus* spp. showed maximum sensitivity to tetracycline (2, 100%) and vancomycin (2, 100%) and maximum resistance to cephalothin, ciprofloxacin, ceftriaxone, amikacin, penicillin, erythromycin, ampicillin, doxycycline, gentamicin, i.e.,(2, 100%). Antibiotic susceptibility pattern of *Staphylococcus aureus* showed that the majority of isolates were sensitive to vancomycin (20, 86.95%) followed by tetracycline (17, 73.91%), norfloxacin (17, 3.91%), doxycycline (17, 73.91%), gentamicin (15, 65.21%), amikacin (13, 56.52%), ceftriaxone (10, 43.47%), chloramphenicol (9, 39.13%), cephalothin (9, 39.13%), ciprofloxacin (6, 26.08%) and penicillin (5, 21.73%). The maximum resistance was observed to penicillin (18, 78.26%) followed by ciprofloxacin (17, 73.91%), ampicillin (17, 73.91%), chloramphenicol (14, 60.86%), cephalothin(14, 60.86%), erythromycin (14, 60.86%), ceftriaxone(13, 56.52%), amikacin (10, 43.47%), gentamicin (8, 34.78%), norfloxacin (6, 26.08%), tetracycline (6, 26.08%) and vancomycin (3, 13.04%). Antibiotic susceptibility pattern of *Enterococcus* spp. showed maximum sensitivity to vancomycin (2, 100%), tetracycline (2, 100%) followed by chloramphenicol (1, 50%), norfloxacin (1, 50%) and maximum resistance to cephalothin (2, 100%), ciprofloxacin (2, 100%), ceftriaxone (2, 100%), amikacin (2, 100%), penicillin (2, 100%), erythromycin (2, 100%), doxycycline (2, 100%), gentamicin (2, 100%) followed by norfloxacin (1, 50%) and chloramphenicol (1, 50%).

**Table 5:** Antibiotic susceptibility test of gram-positive organisms isolated from wound infection %: percentage, µg: micrograms

Antibiotic	*Coagulase positive Staphylococcus * (23)		*Enterococcus* spp. (2)	
	Sensitive	Resistant	Sensitive	Resistant
Chloramphenicol (30µg)	09 (39.13%)	14 (60.86%)	01 (50%)	01 (50%)
Cephalothin (30µg)	09 (39.13%)	14 (60.86%)	0	02 (100%)
Ciprofloxacin (5µg)	06 (26.08%)	17 (73.91%)	0	02 (100%)
Ceftriaxone (30µg)	10 (43.47%)	13 (56.52%)	0	02 (100%)
Amikacin (30µg)	13 (56.52%)	10 (43.47%)	0	02 (100%)
Penicillin (10IU)	05 (21.73%)	18 (78.26%)	0	02 (100%)
Erythromycin (15µg)	09 (39.13%)	14 (60.86%)	0	02 (100%)
Ampicillin (10µg)	06 (26.08%)	17 (73.91%)	0	02 (100%)
Doxycycline (30µg)	17 (73.91%)	06 (26.08%)	0	02 100%)
Gentamicin (10µg)	15 (65.21%)	08 (34.78%)	0	02 (100%)
Norfloxacin (10µg)	17 (73.91%)	06 (26.08%)	01 (50%)	01 (50%)
Tetracycline (30µg)	17 (73.91%)	06 (26.08%)	02 (100%)	0
Vancomycin (30µg)	20 (86.95%)	03 (13.04%)	02 (100%)	0

Table 6 shows the antibiotic sensitivity pattern of gram-negative organisms isolated from wound infection. The majority of *E.coli* strains* *were sensitive to doxycycline (14, 93.33%), followed by gentamicin (13, 86.66%) and tigecycline (12, 80%). *Klebsiella pneumoniae* showed maximum sensitivity to piperacillin/tazobactam (7, 50%), tigecycline (6, 42.85%), and doxycycline (6, 42.85%). *Klebsiella oxytoca* showed maximum sensitivity to piperacillin/tazobactam (4, 80%), netillin (3, 60%), gentamicin (3, 60%) and doxycycline (3, 60%). *Klebsiella aerogenes* showed maximum sensitivity to cephalothin (1, 100%), amoxyclav (1, 100%), netillin 1(100%), norfloxacin (1, 100%), doxycycline (1, 100%), chloramphenicol (1, 100%), piperacillin/tazobactam (1, 100%) and tigecycline (1, 100%). *Pseudomonas aeruginosa* showed maximum sensitivity to chloramphenicol (13, 76.47%) and amoxyclav (13, 76.47%). *Acinetobacter spp*. showed maximum sensitivity to tigecycline (8, 38.09%), cephalothin (5, 23.80%) and amoxyclav (5, 23.80%). *Proteus* spp. showed maximum sensitivity to ceftriaxone (2, 40%), ampicillin (2, 40%), cephalothin (2, 40%), amoxyclav (2, 40%), netillin(2, 40%), gentamicin (2, 40%), norfloxacin (2, 40%), doxycycline (2, 40%), chloramphenicol (2, 40%), piperacillin/tazobactam (2, 40%) and cefepime(2, 40%).

**Table 6:** Antibiotic sensitivity pattern of gram-negative organisms isolated from wound infection *E.coli: Escherichia coli; K. pneumoniae: Klebsiella pneumoniae; K. oxytoca: Klebsiella oxytoca; K. aerogens: Klebsiella aerogens; Ps.aruginosa: Pseudomonas aeruginosa; Acinetobacter *spp*.: Acinetobacter* species

Antibiotic	*E. coli *(15)	*K. pneumoniae *(14)	*K. oxytoca *(05)	*K. aerogenes *(01)	*Ps. aeruginosa* (17)	*Acinetobacter spp.* (21)	*Proteus mirabilis *(05)
Ceftriaxone	06 (40%)	03 (21.42%)	02 (40%)	0	12 (70.58%)	02 (9.52%)	02 (40%)
Ampicillin	05 (33.33%)	02 (14.28%)	02 (40%)	0	12 (70.58%)	03 (14.28%)	02 (40%)
Cephalothin	10 (66.66%)	04 (28.57%)	02 (40%)	01 (100%)	12 (70.58%)	05 (23.80%)	02 (40%)
Amoxyclav	11 (73.33%)	05 (35.71%)	03 (60%)	01 (100%)	13 (76.47%)	05 (23.80%)	03 (60%)
Netillin	12 (80%)	04 (28.57%)	03 (60%)	01 (100%)	12 (70.58%)	03 (14.28%)	02 (40%)
Gentamicin	13 (86.66%)	06 (42.85%)	03 (60%)	0	10 (58.82%)	02 (9.52%)	02 (40%)
Norfloxacin	9 (60%)	03 (21.42%)	01 (20%)	01 (100%)	11 (64.70%)	03 (14.28%)	02 (40%)
Doxycycline	14 (93.33%)	06 (42.85%)	01 (60%)	01 (100%)	14 (82%)	03 (14.28%)	02 (40%)
Chloramphenicol	10 (66.66%)	05 (35.71%)	03 (60%)	01 (100%)	13 (76.47%)	02 (9.52%)	02 (40%)
Piperacillin/ Tazobactam	11 (73.33%)	07 (50%)	04 (80%)	01 (100%)	09 (52.94%)	02 (9.52%)	02 (40%)
Tigecycline	12 (80%)	06 (42.85%)	02 (40%)	01 (100%)	04 (23.52%)	08 (38.09%)	0
Cefepime	09 (60%)	02 (14.28%)	02 (40%)	0	09 (52.94%)	01 (4.76%)	02 (40%)

Table 7 shows the antibiotic resistance pattern of gram-negative organisms isolated from wound infection. *E. coli *showed maximum resistance to ampicillin (10, 66.66%), ceftriaxone (9, 60%), and cefepime (9, 60%). *K. pneumoniae* showed maximum resistance to ampicillin(12, 85.71%), cefepime (12, 85.71%), and ceftriaxone (11, 78.57%). *K. oxytoca* showed maximum resistance to norfloxacin (4, 80%) and doxycycline (4, 80%). *K. aerogenes* showed maximum resistance to ceftriaxone (1, 100%), ampicillin (1, 100%), gentamicin (1, 100%), and cefepime (1, 100%). *Pseudomonas aeruginosa* showed maximum resistance to tigecycline (13, 76.47%), cefepime (8, 47.05%), and piperacillin/tazobactam (8, 47.05%). *Acinetobacter* spp. showed maximum resistance to cefepime (15, 71.42%), piperacillin/tazobactam (14, 66.66%), and chloramphenicol (14, 66.66%). *Proteus mirabilis* showed maximum resistance to tigecycline (5, 100%), ceftriaxone (3, 60%), ampicillin (3, 60%), cephalothin (3, 60%), netillin (3, 60%), gentamicin (3, 60%), norfloxacin (3, 60%), doxycycline (3, 60%), chloramphenicol (3, 60%), piperacillin/tazobactam (3, 60%) and cefepime (3, 60%).

**Table 7:** Antibiotic resistance pattern of gram-negative organisms isolated from wound infection *E.coli: Escherichia coli; K. pneumoniae: Klebsiella pneumoniae; K. oxytoca: Klebsiella oxytoca; K. aerogens: Klebsiella aerogens; Ps.aruginosa: Pseudomonas aeruginosa; Acinetobacter spp.: Acinetobacter* species

Antibiotics	*E. coli *(15)	*K. pneumoniae* (14)	*K. oxytoca* (05)	*K. aerogenes(* 01)	*Ps. aeruginosa* (17)	*Acinetobacter* spp. (21)	*Proteus mirabilis* (05)
Ceftriaxone	09(60%)	11(78.57%)	03(60%)	01(100%)	05(29.41%)	14(66.66%)	03 (60%)
Ampicillin	10(66.66%)	12(85.71%)	03(60%)	01(100%)	05(29.41%)	13(61.90%)	03(60%)
Cephalothin	05(33.33%)	10(71.42%)	03(60%)	0	05(29.41%)	11(52.38%)	03(60%)
Amoxiclav	04(26.66%)	09(64.28%)	02(40%)	0	04(23.52%)	11(52.38%)	02(40%)
Netillin	03(20%)	10(71.42%)	02(40%)	0	05(29.41%)	13(61.90%)	03(60%)
Gentamicin	02(13.33%)	08(57.14%)	02(40%)	01(100%)	07(41.17%)	14 (66.66%)	03(60%)
Norfloxacin	06(40%)	11(78.57%)	04(80%)	0	06(35.29%)	13(61.90%)	03(60%)
Doxycycline	01(6.66%)	08(57.14%)	04(80%)	0	03(17.64%)	12(57.14%)	03(60%)
Chloramphenicol	05(33.33%)	09(64.28%)	02(40%)	0	04(23.52%)	14(66.66%)	03(60%)
Piperacillin/ Tazobactam	04(26.66%)	07(50%)	01(20%)	0	08(47.05%)	14(66.66%)	03(60%)
Tigecycline	03(20%)	08(57.14%)	03(60%)	0	13(76.47%)	08(38.09%)	05(100%)
Cefepime	06(60%)	12(85.71%)	03(60%)	01 (100%)	08(47.05%)	15(71.42%)	03(60%)

## Discussion

Wounds that show persistent inflammation, inadequate remodeling, and unsuccessful re-epithelialization do not heal. In this study, the percentage of wound infection was found to be 41% in the age group of 21-40 years, 29% in the age group of 41-60 years, and 23% in the group of >60 years. The wound infection rate in the 21-40 age group was 41% higher than that of the remaining age groups, and this correlates with the study conducted by Gill et al. [6], whereas a higher number of infected wounds was demonstrated in a younger age group, according to the study by Tarana et al., [7].

Our results demonstrate a predominance of isolated gram-negative organisms (75, 75%) as compared to isolated gram-positive organisms (25, 25%), comparable to other published studies. Most of the studies showed a predominance of gram-negative organisms. Basu et al. [8] showed 39% gram-negative isolates, followed by Kaur Gill et al. [6] with 70.76% gram-negative isolates, and Sowmya et al. showed 60% gram-negative isolates [9]. Our study is analogous to the findings of Sundharan et al., where gram-positive organisms were 31.6% and gram-negative were 68.3% [10]. In contrast, in the study conducted by Roy et al., gram-positive bacteria were 60%, and gram-negative were 40% [11].

From gram-positive organisms, *Staphylococcus aureus* (23%) was the most common organism isolated, followed by *Enterococcus* spp.(2%). This study is similar to the study conducted by Bhalchandra et al. [12], where the predominance of *Staphylococcus aureus* was seen, followed by *Enterococcus* spp. It was observed that the studies conducted by N. Sowmya et al., Bhalchandra et al., and the study conducted by Rao et al. [13] showed only *Staphylococcus aureus *among the gram-positive* *organisms, whereas, in the present study, two types of gram-positive species were isolated, *Staphylococcus aureus* and *Enterococcus* spp. 

In the present study, *Klebsiella* spp. (20%) was the most common organism, followed by *Pseudomonas aeruginosa *(17%), *Acinetobacter* spp. (16%), *E.* *coli* (15%), *Proteus mirabilis* (5%) and *Enterobacter* spp. (2%). This is similar to the study published by Sreekanth et al., where *Klebsiella *and *Pseudomonas aeruginosa *were* *19% and 7.37%, respectively, and were found to be more common [14]. Among gram‑positive isolates, *Staphylococcus* infections were more common. The study conducted by Mundhada et al. showed the most common isolate was *Klebsiella pneumoniae* (34.40%), followed by *Pseudomonas aeruginosa (*23.94%), *Staphylococcus aureus (*22.94%), *Escherichia coli (*7.34%), *Acinetobacte*r spp. (2.75%), *Proteus mirabilis* (2.75%)[15]. Kaur Gill et al. reported that* E. coli *was found to be 29.23%, followed by *Klebsiella* spp. (12.3%), *Enterobacter* spp. (4.92%), *Pseudomonas aeruginosa *(11.12%), *Acinetobacter *spp.(8.66%) and *Proteus mirabilis* (3.14%) [6]. Rao et al. reported *E. coli* (14.02%), *Klebsiella* spp. (12.15%), *Pseudomonas aeruginosa* (21.49%) and *Proteus mirabilis* (7.47%) [11].

Several limitations were encountered in the study. Firstly, the sample size of 100 did not fully represent the diverse population affected by such infections, potentially skewing results. Additionally, variations in wound types and patient comorbidities could introduce confounding factors that affect microbial profiles. Finally, reliance on laboratory techniques may overlook the complexity of in vivo interactions between pathogens and host defenses. These limitations highlight the need for further research to validate findings and improve clinical outcomes.

## Conclusions

The present study emphasizes the burden of wound infections and the determination of antibiotic susceptibility patterns in managing these infections. It is crucial for clinicians to be aware of the bacteriological profile of wound infections, as they represent a significant cause of mortality and morbidity. In this study, *Staphylococcus aureus* was identified as the most common bacterial isolate causing wound infections, followed by *Klebsiella *spp. Antimicrobial susceptibility tests revealed that vancomycin was effective against *Staphylococcus aureus*, followed by tetracycline, while piperacillin/tazobactam and tigecycline showed effectiveness against *Klebsiella *spp*.* The future of wound infection management lies in a multifaceted approach that integrates microbiological research, advanced diagnostic technologies, and personalized treatment plans.
